# Evaluation of an Intravenous Acetaminophen Protocol in the Emergency Department

**DOI:** 10.7759/cureus.52934

**Published:** 2024-01-25

**Authors:** Aaron B Deutsch, John D DelBianco, Patrick Fagan, Kimberly Sharpe, Jason Laskosky, Laura Koons, Gillian A Beauchamp, Kenneth D Katz

**Affiliations:** 1 Department of Emergency and Hospital Medicine, Lehigh Valley Health Network/University of South Florida (USF) Morsani College of Medicine, Allentown, USA; 2 Department of Emergency and Hospital Medicine, Division of Medical Toxicology, Lehigh Valley Health Network/University of South Florida (USF) Morsani College of Medicine, Allentown, USA; 3 Department of Pharmacy, Lehigh Valley Health Network/University of South Florida (USF) Morsani College of Medicine, Allentown, USA

**Keywords:** patient safety, opioid epidemic, parenteral analgesia, emergency department analgesia, intravenous acetaminophen

## Abstract

Background: Acute pain is a leading reason for Emergency Department (ED) evaluation, accounting for nearly half of all ED visits. Therefore, providing effective non-opioid analgesics in the ED is critical. Oral acetaminophen (APAP) is commonly administered in the ED but is limited to patients tolerating oral intake. Intravenous (IV) APAP provides significant pain reduction parenterally. The purpose of this quality assessment project was to evaluate the frequency of opioid use in patients receiving IV APAP, the safety of IV APAP, and compliance with an ED IV APAP protocol.

Methods: This study included all patients who received IV APAP in the ED of a tertiary care, level I trauma center, during a three-month period. The protocol required ED patients to be NPO (nil per os), 18 years or older, and administered with a single 1000 mg dose. The adverse reactions within 24 hours post-IV APAP, ED length of stay (LOS), and opioid administration within four hours post-IV APAP were assessed.

Results: Ninety-four patients received IV APAP. All patients received a 1000 mg dose. One patient received more than one dose, but this patient had a 22-hour ED LOS. Two patients received oral medications within one hour of IV APAP (one received an antacid, and the other received carbamazepine and lamotrigine). An opioid was administered to 22 of the 94 (23.4%) patients during the four-hour protocol period. There were no reports of adverse reactions.

Conclusions: The results show excellent compliance with the protocol. IV APAP was safe and well-tolerated. Notably, most patients did not receive an opioid within four hours of IV APAP. IV APAP can be safely and effectively utilized as an analgesic and lessen ED opioid use.

## Introduction

Acute pain is a leading cause of presentation to the Emergency Department (ED) [1]. In 2020, there were approximately 131 million ED visits, with one study showing that about 42% of ED visits are related to pain [1,2]. Patient co-morbidities may limit pain-management options, requiring consideration of alternative modalities. For example, nonsteroidal anti-inflammatory medications (NSAIDs) are contraindicated in patients with acute or chronic kidney disease [3]. Other factors, such as pregnancy and the ability to tolerate oral medications, can further limit the pharmacological options to treat pain [4,5].

Acetaminophen is a common analgesic used in the ED. Intravenous acetaminophen (IV APAP) provides a significant reduction in both pain intensity and time to meaningful pain improvement [6]. Published data generally have not proven IV APAP superior to oral APAP, although IV APAP remains an option when other routes of administration are not viable [7,8]. A randomized, placebo-controlled trial by Bektas et al. found similar efficacy between IV APAP and IV morphine for treating renal colic [9]. These findings suggest that IV APAP may be beneficial for treating pain in the ED and could serve as an alternative to IV opioids for parenteral analgesia when NSAIDs are contraindications.

Both patients and clinicians may have concerns about opioid use, given there were over 80,000 opioid-associated deaths in the United States in 2021, according to the National Center for Health Statistics (NCHS) [10]. The rising incidence of opioid use disorder and overdose deaths involving opioids has been with both illicit and prescribed opioids, with patients being almost twice as likely to use opioids in the future after an initial opioid prescription [11]. Therefore, non-opioid treatment for pain is an important consideration. Alternatives to opioids, such as IV APAP, may be utilized to decrease the frequency and number of opioids required in the ED.

This study describes a hospital-implemented protocol for the use of IV APAP in the ED. The percentage of patients receiving opioids in addition to IV APAP, adverse events related to IV APAP, and ED provider compliance with the protocol were evaluated.

This article was previously presented in part as an abstract at the Pennsylvania College of Emergency Physicians Scientific Assembly in Pocono Manor, Pennsylvania, United States, on May 5, 2023.

## Materials and methods

Study design

This study included all patients who received IV APAP during a three-month period in the ED of Lehigh Valley Health Network, Allentown, Pennsylvania, United States, a tertiary care, level I trauma center. The protocol required patients to be NPO (nil per os), 18 years or older, and administered a single 1000 mg IV dose. This quality assessment project was designated as nonhuman subjects research by the Institutional Review Board. This study was approved by the Lehigh Valley Health Network Institutional Review Board (approval number: STUDY000001169).

Data collection

The adverse reactions within 24 hours post-IV APAP, ED length of stay (LOS), oral medication administration within one hour post-IV APAP, and opioid administration within four hours post-IV APAP were tracked. Cases were retrieved from the electronic medical record via standard pharmacy processes based on orders for IV APAP. Cases of IV APAP administration were documented per standard hospital pharmacy protocol, de-identified, and stored in a password-protected spreadsheet on a secure drive within a secure server.

## Results

Ninety-four patients received IV APAP as first-line treatment for acute pain. All patients received a 1000 mg dose. Protocol adherence was observed in 91 of the 94 (96.8%) cases, with the remaining three cases involving protocol violations (Table 1). An opioid was administered to 22 of the 94 (23.4%) patients during the four-hour protocol period. There were no reports of adverse reactions, side effects, or incorrect dosing administrations.

**Table 1:** Protocol violations IV APAP: Intravenous acetaminophen; ED: Emergency department; LOS: Length of stay

Cases Violating Protocol	Nature of Violation
Patient A	Received two doses of IV APAP (had 22-hour ED LOS)
Patient B	Received oral medication (aluminum-magnesium hydroxide with simethicone) within one hour of IV APAP
Patient C	Received oral medication (carbamazepine and lamotrigine) within one hour of IV APAP

## Discussion

This study demonstrates that ED clinicians complied with the IV APAP protocol as the first-line therapy for acute pain. There were no reports of adverse reactions. Notably, only 23.4% of the patients received an opioid within four hours of IV APAP. In comparison, according to data from the NCHS, an opioid was given or prescribed for 30.9% of pain-related ED visits nationally [12]. It may represent an opioid-sparing effect with the use of IV APAP, which is consistent with studies that have evaluated the efficacy of IV APAP in the postoperative setting [13,14]. However, IV APAP may not completely supplant opioid use as other studies find that IV APAP alone is less effective than opioids and does not augment the analgesic effects of opioids [15,16].

Nevertheless, decreasing opioid use is an important goal for both patient safety and economic reasons. Opioids are associated with substantial adverse effects, including constipation, nausea, vomiting, and potentially fatal respiratory depression [17]. Opioid overdose deaths may involve both illicit substances and prescribed medications; one study demonstrated that 36% of patients with opioid use disorder had at least one opioid prescription within the past year [18]. Additionally, studies show that patients are almost twice as likely to use opioids in the future after receiving an initial prescription [11]. IV APAP, in contrast, has largely minor adverse effects [13]. Although there were no reports of adverse reactions in this study, IV APAP has been associated with hypotension in severely ill patients [19]. IV APAP dosing errors, some resulting in hepatic injury, have occurred. These errors are more common in the pediatric population because of the calculations required with weight-based dosing. Experts recommend approaching IV APAP overdose in the same manner as an oral APAP overdose, administering N-acetylcysteine when treatment is indicated, according to the Rumack-Matthew nomogram [20,21].

Opioid use also takes an economic toll. It has been estimated that the cost to society of harmful prescription opioids is over $8.6 billion [22]. Moreover, postoperative opioid prescriptions are associated with higher costs [23]. Some institutions have implemented cost-saving initiatives to reduce IV APAP use in the inpatient setting; however, compared to opioid monotherapy, IV APAP reduces hospital costs without any significant difference in pharmaceutical costs [24,25]. IV APAP was associated with fewer side effects and adverse drug events for patients, which may further reduce healthcare costs [14,26].

Emergency medicine clinicians treat a diverse patient population with complex medical histories and co-morbidities, which may limit pharmaceutical options for pain relief. For example, clinicians often avoid NSAIDs for patients with kidney disease, coronary artery disease, history of gastrointestinal hemorrhage, or those currently taking anticoagulant medications [27]. Pregnancy is another condition commonly encountered in the ED that requires careful consideration regarding analgesics. The American College of Obstetricians and Gynecologists (ACOG) has identified APAP as one of the safe pain relievers for pregnant individuals [28]. Nausea and vomiting are common symptoms in pregnancy. The utility of IV APAP in individuals who cannot tolerate oral intake, particularly in pregnancy, seems beneficial [4,29].

Limitations of this study include that it was retrospective and conducted at one hospital system, limiting the generalizability of the results. Additionally, this study excluded those under the age of 18.

## Conclusions

This study demonstrates the successful implementation of an IV APAP protocol in the ED. Nearly all cases maintained protocol compliance. IV APAP was found to be safe, with no adverse effects reported. In addition, most patients receiving IV APAP did not also receive opioids.
